# Patient Outcomes and Management Strategies for Intensive Care Unit (ICU)-Associated Delirium: A Literature Review

**DOI:** 10.7759/cureus.61527

**Published:** 2024-06-02

**Authors:** Ahmed M Abdelbaky, Mohamed S Eldelpshany

**Affiliations:** 1 Intensive Care Unit, Rashid Hospital, Dubai Health, Dubai, ARE

**Keywords:** cognitive impairment, icu, icu psychosis, critical care medicine, icu delirium

## Abstract

Delirium is a significant public health concern, with tremendous implications for patient outcomes. Intensive care unit (ICU)-related delirium is gaining attention due to the higher prevalence of delirium in ICU-admitted patients. The most common negative outcomes of ICU delirium include cognitive impairments, functional dependence, high incidence of mortality, extended stay in the ICU, and high costs. So far, no single etiological factor has been identified as the sole cause of delirium. Several functional, neurotransmitter, or injury-causing hypotheses have been proposed for ICU delirium. Several risk factors contribute to the development of delirium in patients admitted to the ICU. These are age, gender, types of sedation, physical restraints, medical and surgical interventions, pain, and extended stay in the ICU. The most commonly used assessment modules for ICU delirium are the PREdiction of DELIRium in ICu patients (PRE-DELIRIC), Early PREdiction model for DELIRium in ICu patients (E-PRE-DELERIC), and Lanzhou Model, Confusion Assessment Method for the Intensive Care Unit (CAM-ICU), Intensive Care Delirium Screening Checklist (ICDSC), and Delirium Rating Scale (DRS). There is no proper treatment for ICU delirium; however, it can be managed through various pharmacological and non-pharmacological interventions. Healthcare providers should receive constant education and training on delirium recognition, prevention, and management to enhance patient care and outcomes in the ICU. Further research is needed on the effective prevention and management of ICU delirium.

## Introduction and background

The intensive care unit (ICU) provides a perfect environment that can either trigger or exacerbate a psychological disorder or neuropsychiatric syndrome. Among various ICU-related disorders such as post-traumatic stress disorder (PTSD) [1], anxiety, and depression [2], delirium is one of the most common behavioral manifestations post-ICU visit [3]. It affects a high proportion of patients in the ICU. Some estimates suggest that delirium can affect over half of the patients admitted to the hospital and 80% of the mechanically ventilated ICU patients [4]. The prevalence of delirium can vary as it is a term that is often used inconsistently with acute brain dysfunction, septic encephalopathy, and ICU syndrome, depending on the location and medical specialty [5]. According to the Diagnostic and Statistical Manual of Mental Disorders (DSM)-5, delirium is described as a disturbance in attention and awareness that develops over a short period of time (usually hours to a few days) and tends to fluctuate in severity during the course of a day. The main cause of disturbance is thought to be a direct physiologic consequence of a medical condition, a medication used, or an intoxicating agent [6]. ICU delirium, a term commonly used to refer to delirium post-ICU visit, is a significant concern in critical patients that can affect their outcomes and actual course of illness [7].

There are different motoric phenotypes of delirium. Hyperactive delirium is characterized by increased motor activity, speech, abnormal content of verbal output, irritability, restlessness, combativeness, and hyperconsciousness. Hypoactive delirium, on the other hand, is characterized by reduced motor activity, alertness, speech, apathy, hypersomnolence, and unawareness. Some patients may also have a mixed type of delirium, characterized by symptoms of hyperactive delirium at times and symptoms of hypoactive delirium at other times [8]. The main risk factors for delirium in the ICU include the need for mechanical ventilation, severity of illness, and age [9]. There is no unifying mechanism for delirium, but there are numerous functional, neurotransmitter, or injury-causing hypotheses. It can be misdiagnosed if a validated diagnostic instrument is not used for its diagnosis. Commonly used screening tools for ICU delirium are the Confusion Assessment Method for the Intensive Care Unit (CAM-ICU) and Intensive Care Delirium Screening Checklist (ICDSC) [10].

There is significant evidence that points towards poor outcomes of delirium in critically ill patients. These outcomes include an increased risk of cognitive impairment, increased length of hospital stay, and ICU mortalities. There is also evidence of long-term adverse consequences of ICU delirium [9]. Furthermore, the severity of adverse health and patient outcomes depends upon the severity of delirium [4]. It is generally accepted that there are no identifiable therapies to decrease the duration of delirium but the underlying causes are managed through pharmacological and non-pharmacological strategies [11]. Despite having a profound impact on patient health and causing severe adverse outcomes, ICU delirium often goes unrecognized [12].

So, it is imperative to delve deeper into the literature surrounding ICU delirium to gain a comprehensive understanding of its impact on patient outcomes. This literature review aims to shed light on the outcomes associated with ICU delirium and highlight the need for effective prevention and management strategies.

## Review

Methodology

The current review was a literature review as we aimed to provide a general summary of the existing literature. To find relevant studies that investigated ICU-related delirium, we searched various databases. The key databases searched for this literature review included PubMed and Web of Science. Furthermore, the reference section of the potential studies and Google Scholar was also explored to enrich the literature review. The search words used included the combination of "intensive care unit," or "ICU," or "critically ill" and "delirium" or "confusion state" or "acute confusion." Further details of all the keywords are provided in Table 1. To ensure that we included a significant amount of evidence on the topic, the search was not limited by study type or duration. The literature review included original studies, review articles, and systemic reviews. All the studies that discussed ICU-related delirium in patients, including its impact on patient outcomes, were included. However, studies that assessed delirium in general were excluded.

**Table 1:** Terms and strategy used for literature search ICU: intensive care unit; Ti: title; Ab: abstract; MeSH: Medical Subject Headings

Keywords used and their combination	
#1 delirium [MeSH]	#9 intensive care units [MeSH]
#2 acute confusional state [Ti/Ab]	#10 ICU [Ti/Ab]
#3 encephalopathy [Ti/Ab]	#11 critical care [Ti/Ab]
#4 organic brain syndrome [Ti/Ab]	#12 critical illness [Ti/Ab]
#5 brain dysfunction [Ti/Ab]	#13 critically ill [Ti/Ab]
#6 confusion state [Ti/Ab]	#14 OR/9-13
#7 acute confusion [Ti/Ab]	#15 8 AND 14
#8 OR/1-7	

Results and discussion

The database search in PubMed and Web of Science revealed 12,561 results (Table 2). Furthermore, research in Google Scholar also revealed around 140 relevant articles. We looked for three main aspects of ICU delirium in studies. First, studies that described patient outcomes were included; however, we included information from the studies that were relevant and covered this particular aspect. We did not include all studies identified. Further studies that discussed screening and management of delirium were also included in the analysis. The details of these three aspects are given in detail below.

**Table 2:** Search strategy ICU: intensive care unit; Ti: title; Ab: abstract; MeSH: Medical Subject Headings

Key variable	Sub-terms	Search options	PubMed	Web of Science
1. Delirium	1.1 delirium	MeSH	13,345	22,120
	1.2 acute confusional state	Ti/Ab	476	539
	1.3 encephalopathy	Ti/Ab	58,329	56,660
	1.4 organic brain syndrome	Ti/Ab	594	931
	1.5 brain dysfunction	Ti/Ab	4080	57,101
	1.6 confusion state	Ti/Ab	31	7,390
	1.7 acute confusion	Ti/Ab	444	3,214
(((((("Delirium"[Mesh]) OR (acute confusional state[Title/Abstract])) OR (encephalopathy[Title/Abstract])) OR (organic brain syndrome[Title/Abstract])) OR (brain dysfunction[Title/Abstract])) OR (confusion state[Title/Abstract])) OR (acute confusion[Title/Abstract])			76,186	143,346
2. Intensive care unit	2.1 intensive care units	MeSH	109,212	153,406
	2.2 ICU	Ti/Ab	90,677	86,996
	2.3 critical care	Ti/Ab	46,088	106,104
	2.4 critical illness	Ti/Ab	14,307	23,910
	2.5 critically ill	Ti/Ab	63,396	60,723
(((("Intensive Care Units"[Mesh]) OR (ICU[Title/Abstract])) OR (critical care[Title/Abstract])) OR (critical illness[Title/Abstract])) OR (critically ill[Title/Abstract])			233,117	324,986
3. Overall	((((((("Delirium"[Mesh]) OR (acute confusional state[Title/Abstract])) OR (encephalopathy[Title/Abstract])) OR (organic brain syndrome[Title/Abstract])) OR (brain dysfunction[Title/Abstract])) OR (confusion state[Title/Abstract])) OR (acute confusion[Title/Abstract])) AND ((((("Intensive Care Units"[Mesh]) OR (ICU[Title/Abstract])) OR (critical care[Title/Abstract])) OR (critical illness[Title/Abstract])) OR (critically ill[Title/Abstract]))		4667	7,894

Impact of ICU Delirium on Patient Outcomes

Cognitive outcomes: Cognitive impairment is one of the most common manifestations of ICU delirium. A longer duration of delirium is associated with worse cognition defects after adjustment for pre-existing cognitive function, age, exposure to sedatives, and severity of illness. Although the cognitive performance of the ICU survivors improves over time in some patients, it may persist in some patients [13]. Previously, various studies have investigated the long-term impairment in ICU delirium patients. In a recent prospective cohort study, it was reported that delirium was independently associated with cognitive impairment at ICU discharge and symptoms of PTSD after 12 months [14]. 

Apart from ICU, some studies have also reported cognitive outcomes after postoperative delirium. A meta-analysis by Huang et al. reported that postoperative delirium has a significant relation with both short-term and long-term worse cognitive outcomes [15]. A significant association of delirium with cognitive decline was also reported in a meta-analysis by Goldberg et al. that included 23 studies on surgical and non-surgical patients [16]. The association of hyperactive and hypoactive delirium was assessed in a secondary analysis of a prospective cohort. The results of this study found that the hypoactive type was more persistent and common than hyperactive delirium (71% versus 17%). This study demonstrated that a longer duration of hypoactive delirium was linked with worse global cognition and executive functioning, while the hyperactive type was not linked to executive functioning and global cognition in critically ill patients [8]. In another study, subsyndromal delirium which is considered an intermediate state between non-delirium and delirium was found to be associated with worse cognitive decline at three months after discharge but was improved after six months [17].

Functional outcomes: Functional outcomes are one of the key measures used in assessing the overall well-being of ICU delirium patients. A prospective control study conducted across three hospitals, involving critically ill patients admitted to surgical ICUs, revealed that postoperative delirium emerged as an independent risk factor linked to the functional outcomes of ICU survivors [18]. A recent follow-up study stated that delirium burden has a long-term association with neurological functional outcomes up to 2.5 years after critical illness [19]. Rengel et al., in their study, reported a statistically significant association between the duration of motoric subtype (hypoactive) delirium and functional outcomes after three months. However, they could not find any evidence of hyperactive delirium association with functional dependence after critical illness [20]. There is also some inconclusive shreds of evidence that the functional ability of the person has links with the severity of delirium after a critical illness [4].

Mortality rates: An acute episode of delirium also has a profound impact on the mortality rates among critically ill patients, but their relationship has not been extensively explored. Sanchez et al., in their study, reported that frail people who experience an episode of delirium in the ICU have a 35% rate of mortality [21]. Similar findings have been shared in a meta-analysis that included 71 studies. It was highlighted that there were significant odds of mortality in patients after an episode of delirium and these odds of mortality were much higher in ICU patients [22].

Another recent population-based retrospective study of adult patients admitted to surgical ICUs investigated long-term outcomes of ICU delirium. The primary outcome of delirium in this study was mortality, and secondary outcomes included readmission to the hospitals and frequent hospital visits. The results of this study showed that delirium was associated with high mortality rates between 0 and 30 days after being discharged from the hospital (hazard ratio: 1.4). However, after 30 days of discharge, there was no significant difference in the mortality rates among the delirium and non-delirium patients (non-delirium: 2.6%; delirium: 3.9%) [9].

A prospective cohort study of critically ill adults revealed that among 1040 critically ill patients, 204 patients died out of the hospital and 214 died in the hospital in one-year follow-up. Delirium was the most common factor among these patients (740 patients) with a median of four (2-7) days. Out of 740 delirium patients, 733 were suffering from hypoactive delirium. Hypoactive delirium subtype was independently associated with an increased risk of death (P=0.03). On the other hand, hyperactive delirium was not associated with an increase in hospital death risk (P=0.19) [23].

Length of ICU stay: Prolonged ICU stays not only increase healthcare costs but also expose patients to the risk of developing other complications linked with extended bed rest and exposure to the ICU environment. Several studies have reliably found that ICU delirium is related to long ICU stays. In a recent review, 41 studies were reviewed for the length of ICU stay for both delirium and non-delirium patients. The mean difference between the stay was found to be significant (4.77 days, P<0.001). Also, the mean difference between the ICU costs between the patients with and without delirium was significant (P<0.001). These results were an indication that ICU delirium is associated with longer ICU stays and hospital costs [24]. Postoperative delirium also has a higher incidence rate, and it is also an independent predictor for an extended length of stay (LOS) in the ICU (P<0.001) in older adults [25]. Another observational prospective study concluded that patients who develop delirium in the ICU have longer hospital and ICU LOS with a higher rate of revisits and readmissions [26]. The negative impact of delirium on the ICU stay was depicted by Ma et al. in their study. They found a significant difference in the days of ICU stay between patients with and without delirium after liver transplantation (P=0.002) [27].

Factors Contributing to the ICU Delirium

There is a variety of factors that are thought to be the contributors to ICU delirium. The most studied factors are given below in Table 3.

**Table 3:** Factors contributing to the development of ICU delirium ICU: intensive care unit

Study and year	Design	Risk factors
Wu et al. (2023) [28]	Systematic review and meta-analysis	Age, gender, physical restraints, pain, surgery, sleep deprivation, and physical restraints
Gravante et al. (2021) [29]	Observational study	Age, gender, medical and surgical interventions
Kooken et al. (2021) [30]	Prospective cohort study	Age, emergency medical or surgical referral, use of physical restraints, and organ failure
Lobo-Valbuena et al. (2021) [26]	Observational prospective study	Age, gender, and organ failure
Rahimi-Bashar et al. (2021) [31]	Prospective cohort	Gender, head trauma, smoking, extended ICU stay, and mechanical ventilation
Li et al. (2020) [32]	Review	Sedatives, hypoxia, extended ICU stay
Jayaswal et al. (2019) [12]	Prospective study	Age, gender, tobacco, chronic liver disease, past history of delirium, fever, hypoxia, raised levels of benzodiazepine, creatinine, and bilirubin
Pan et al. (2019) [33]	Case-control study	Sedatives, physical restraints, and ICU stay

Assessment, Prevention, and Management of ICU Delirium

Delirium is an underdiagnosed public health problem with huge societal downstream impacts and is missed 75% of the time by healthcare professionals. Delirium severity is not measured often due to the lack of validated delirium assessment tools. The most commonly used assessment methods are discussed below.

PREdiction of DELIRium in ICu patients (PRE-DELIRIC), Early PREdiction model for DELIRium in ICu patients (E-PRE-DELERIC), and Lanzhou Model: These three models can aid in preventing and treating delirium. The first model comprises 10 predictors, the second model consists of nine predictors, and the third model comprises 11 predictors for the prediction of ICU-related delirium [10]. A study by Linkaitė et al. investigated the PRE-DELIRIC model for the diagnosis of ICU delirium. Their findings showed that the PRE-DELIRIC model was able to predict delirium in the patients within 24 hours of admission to the ICU [34]. Similarly, a retrospective study by Liang et al. also reported that PRE-DELIRIC has high predictive value in ICU patients. Their study included 375 ICU patients [35]. The efficacy of the Lanzhou Model by Chen et al. has also been validated previously, with results supporting the use of this model in clinical practice to identify ICU delirium [36]. 

CAM-ICU: CAM-ICU consists of acute onset of mental status change, cognitive disturbance, inattention, and altered levels of consciousness. CAM-ICU-7 is also a validated scale for measuring the severity of delirium. This scale ranges from 0 to 7 scores. Scores from 0 to 2 are linked with no delirium, mild and moderate delirium is considered upon scores 3-5, and severe delirium is reported when the scale scores are 6 or 7. A systemic review by Miranda et al. aimed to evaluate the predictive efficacy of CAM-ICU in adult patients in ICU [37]. They included a total of 25 studies in their systemic review. Their findings showed that CAM-ICU has a pooled sensitivity of 78% and specificity of 95% [37]. In another systemic review and meta-analysis, Gusmao-Flores et al. reported that CAM-ICU has a sensitivity and specificity of 80.0% and 95.9%, respectively [38].

ICDSC: This checklist consists of eight items all presenting from score 1, resulting in a total score that ranges from 0 to 8. These scores are categorized as follows: 0 score: no delirium, 1-3 score: subsyndromal delirium, and 4 and higher score, delirium. Boßelmann et al. in their prospective study investigated the efficacy of ICDSC for delirium. Their findings showed that ICDSC has a sensitivity and specificity of 98% and 55%, respectively, for screening delirium [39]. Similarly, a cross-sectional study by Krewulak et al. reported that ICDSC is a better predictor of delirium outcomes compared to CAM-ICU-7 [40]. A systemic review and meta-analysis also reported that the pooled sensitivity of ICDSC was 74% whereas its pooled specificity was 81.9%. Their review included 13 studies, with four studies using ICDSC [38].

Delirium Rating Scale (DRS): Delirium experts also use DRS-R98 for the assessment of delirium and its severity. It is a 13-item scale and includes symptoms like hallucinations, mood lability, wake, and sleep cycle disturbances, thinking abnormalities, motor retardation, agitation, attention impairments, etc. [41]. The complexity of this scale poses a challenge due to the need for trained professionals to administer it, leading to occasional variations in results. de Negreiros et al. have previously conducted studies to validate the DRS-R-8 for Portuguese. Their findings showed that the DRS scale has a sensitivity and specificity of 92.6% and 94.6%, respectively [42]. Similarly, Almuhairi et al., in their study, also showed that DRS has an excellent validity and internal consistency in critical care [43].

Pharmacological Management

There are several pharmacological options available for the treatment of ICU delirium. Among various options, antipsychotics are the most popular pharmacological choice. Antipsychotics can be used for the short-term control of severe agitation or aggressive behavior of the patient towards the ICU team and anxiety [10]. Swan et al., in their study, reported that antipsychotics are administered to one in every 10 ICU patients. However, there are certain risks associated with the use of antipsychotics. For example, if they are administered in the absence of a documented mental disorder, they can lead to higher ICU, hospital LOS, and mortality risk [44]. Once delirium has developed, only supportive treatment is an option. Morphine, dexmedetomidine, and quetiapine have been found to be effective against ICU delirium [45]. A study by Devlin et al. reported that quetiapine when combined with haloperidol leads to faster resolution of delirium and improves functional outcomes [46]. Apart from these, oral alpha-2 agonists such as guanfacine and anticonvulsant medications like valproic acid have been explored as potential pharmacological options following early studies indicating a possible reduction in both the duration and severity of delirium [47,48]. However, larger randomized studies and systematic reviews have failed to demonstrate any significant benefits of antipsychotic medications or oral alpha-2 agonists in reducing the occurrence or duration of delirium [49,50]. Consequently, current practice guidelines do not recommend their routine use for delirium treatment [51]. Despite this, these medications are often prescribed in clinical settings, especially in managing hyperactive symptoms in ICU delirium to control patient agitation or facilitate patient care. A pragmatic trial to investigate the efficiency of a pharmacological management bundle to manage and treat ICU delirium showed that the pharmacological bundle was not effective in reducing the duration of delirium and its severity among critically ill patients [52]. This variability of the outcomes proves the need for a large-scale assessment to further examine the role of pharmacological management of ICU delirium. However, in the case of pharmacological management, the most important thing to keep in mind is the cause of the delirium. 

Non-pharmacological Interventions

When no pharmacological agent shows a significant impact on delirium, bundling it with non-pharmacological strategies is also becoming the mainstay of ICU care. There is moderate certainty evidence that multicomponent non-pharmacological interventions which target various risk factors for delirium have reduced the incidence of ICU delirium by 43%. However, mortality rates are not affected by this intervention [53]. Future research should be focused on contrasting different multicomponent programs for the selection of the most useful interventions [54]. Lange et al., in their review, reported that multicomponent non-pharmacological interventions show promise in the treatment of ICU delirium. Light therapy is particularly helpful in the treatment of ICU delirium [55]. A recent study comparing the effectiveness of pharmacological and non-pharmacological management to prevent ICU delirium failed to provide a piece of firm evidence about the most effective method. Hence, more research is needed on targeted interventions to lower and prevent the incidence of ICU delirium [56].

Future directions and implications

There is a gap in the literature concerning the effectiveness of different management strategies for ICU delirium and their influence on outcomes, warranting further investigation. Clinicians should prioritize routine delirium screening and assessment in the ICU for the on-time identification and management of delirious patients, thus potentially improving outcomes. Furthermore, combining clinical methods with paraclinics is the fundamental pillar in medical conduct in the face of delirium. Healthcare providers should receive constant education and training on delirium recognition, prevention, and management to enhance patient care and outcomes in the ICU. Hospitals should focus on quality improvement initiatives aimed at delirium prevention, such as the integration of delirium protocols and regular auditing of delirium management practices in the ICU.

## Conclusions

This literature review showed that ICU delirium is associated with negative outcomes including longer hospital stays, increased mortality rates, higher healthcare costs, a greater risk of long-term cognitive impairment, longer duration of mechanical ventilation, and overall longer ICU and hospital stays. These outcomes highlight the importance of early detection and management of delirium in the ICU to improve patient outcomes and reduce mortality rates. Healthcare professionals should prioritize delirium screening and interventions in the critical care setting to optimize patient care and enhance recovery outcomes. Future research focused on implementing evidence-based strategies in clinical practice and emphasizing delirium prevention is needed to enhance patient outcomes and quality of care in the ICU setting.
